# Suicides Among American Indian/Alaska Natives — National Violent Death Reporting System, 18 States, 2003–2014

**DOI:** 10.15585/mmwr.mm6708a1

**Published:** 2018-03-02

**Authors:** Rachel A. Leavitt, Allison Ertl, Kameron Sheats, Emiko Petrosky, Asha Ivey-Stephenson, Katherine A. Fowler

**Affiliations:** ^1^Oak Ridge Institute for Science and Education, Oak Ridge Associated Universities; ^2^Division of Violence Prevention, National Center for Injury Prevention and Control, CDC.

Suicide disproportionately affects American Indians/Alaska Natives (AI/AN). The suicide rate among AI/AN has been increasing since 2003 (*1*), and in 2015, AI/AN suicide rates in the 18 states participating in the National Violent Death Reporting System (NVDRS) were 21.5 per 100,000, more than 3.5 times higher than those among racial/ethnic groups with the lowest rates.* To study completed suicides across all ages of AI/AN, NVDRS data collected from 2003 to 2014 were analyzed by comparing differences in suicide characteristics and circumstances between AI/AN and white decedents. Group differences were assessed using chi-squared tests and logistic regression. Across multiple demographics, incident characteristics, and circumstances, AI/AN decedents were significantly different from white decedents. More than one third (35.7%) of AI/AN decedents were aged 10–24 years (versus 11.1% of whites). Compared with whites, AI/AN decedents had 6.6 times the odds of living in a nonmetropolitan area, 2.1 times the odds of a positive alcohol toxicology result, and 2.4 times the odds of a suicide of a friend or family member affecting their death. Suicide prevention efforts should incorporate evidence-based, culturally relevant strategies at individual, interpersonal, and community levels (*2*) and need to account for the heterogeneity among AI/AN communities (*3*,*4*).

CDC’s NVDRS is an active state-based surveillance system that monitors the occurrence and characteristics of violent deaths, including suicides. NVDRS links three data sources (death certificates, coroner/medical examiner reports, and law enforcement reports) to create a comprehensive picture of who dies from violence, where and when victims are injured, and what factors contributed to the victim’s death. This report includes all available 2003–2014 NVDRS data from the 18 participating states.^†^ Analyses were limited to suicide decedents aged ≥10 years. Non-Hispanic AI/AN are defined in NVDRS as persons with ancestries of the original inhabitants of North America who maintain their cultural identification.^§^^,^^¶^ Non-Hispanic whites (whites) were used as a comparison group because they have the second-highest suicide rate, but concentrated among different age groups than AI/AN, allowing for comparisons that might reveal unique contributors to suicide above general risk factors. Rural-Urban Commuting Area codes were used to classify geographic areas into metropolitan and nonmetropolitan categories.** Demographics, incident characteristics, and precipitating circumstances were examined by race/ethnicity using chi-squared tests. Significant chi-squared results (p<0.05) were further examined using logistic regression, controlling for age and sex.

From 2003 to 2014, a total of 1,531 suicides among AI/AN and 103,986 among whites were collected in NVDRS (Table 1). More than one third (35.7%) of AI/AN suicides occurred among youths aged 10–24 years (9.8% aged 10–17 years, 25.9% aged 18–24 years). In contrast, 11.1% of suicides among whites were in persons aged 10–24 years (2.5% aged 10–17 years, 8.6% aged 18–24 years). More than two thirds (69.4%) of AI/AN decedents resided in nonmetropolitan areas, whereas the majority of white decedents (72.7%) resided in metropolitan areas (adjusted odds ratio [aOR] = 6.6; 95% confidence interval [CI] = 5.9–7.3). The largest proportion of both AI/AN and white decedents died by firearm (42.1% and 52.9%, respectively), with hanging/strangulation/suffocation being the next largest proportion (39.7% and 22.5%, respectively).

**TABLE 1:** Selected demographic and incident characteristics of non-Hispanic American Indian/Alaska Natives and non-Hispanic white suicide decedents — National Violent Death Reporting System, 18 states,* 2003–2014

Characteristic	No. (%)		aOR (95% CI)^†^
	AI/AN (N = 1,531)	White (N = 103,986)	
**Age group (yrs)**			
10–17**^§^**	150 (9.8)	2,554 (2.5)	—
18–24**^§^**	396 (25.9)	8,958 (8.6)	—
25–44**^§^**	665 (43.4)	33,550 (32.3)	—
45–64**^§^**	279 (18.2)	41,428 (39.8)	—
≥65**^§^**	41 (2.7)	17,404 (16.8)	—
**Sex**			
Male	1,190 (77.7)	80,798 (77.7)	—
Female	341 (22.3)	23,184 (22.3)	—
**Nonmetropolitan resident^¶^**			
Nonmetropolitan**^§^**	1,063 (69.4)	27,665 (27.3)	6.6 (5.9–7.3)
**Mechanism**			
Firearm**^§^**	645 (42.1)	55,035 (52.9)	0.8 (0.7–0.9)
Hanging, strangulation, suffocation**^§^**	607 (39.7)	23,358 (22.5)	1.6 (1.4–1.7)
Poisoning**^§^**	175 (11.4)	18,508 (17.8)	0.7 (0.6–0.8)
Motor vehicle**^§^**	41 (2.7)	1,220 (1.2)	1.7 (1.2–2.3)
Sharp instrument	24 (1.6)	1,895 (1.8)	—**
Fall**^§^**	12 (0.8)	1,699 (1.6)	—^††^
Other (single method)	19 (1.2)	1,528 (1.5)	—**
**Location**			
House or apartment**^§^**	1,124 (73.4)	78,360 (75.4)	1.0 (0.9–1.1)
Transport related^§§^	112 (7.3)	8,529 (8.2)	—**
Natural area**^§^**^,¶¶^	98 (6.4)	4,670 (4.5)	1.3 (1.1–1.6)
Supervised facility**^§^**^,^***	45 (3.0)	1,567 (1.5)	1.6 (1.2–2.1)
Hotel/Motel	24 (1.6)	2,351 (2.3)	—**
Abandoned building or industrial setting**^§^**^,†††^	19 (1.2)	439 (0.4)	—^††^
School including college**^§^**	12 (0.8)	192 (0.2)	—^††^
Other	83 (5.4)	5,875 (5.6)	—**

**Abbreviations:** AI/AN = non-Hispanic American Indian/Alaska Native; aOR = adjusted odds ratio; CI = confidence interval; white = non-Hispanic white.

* Alaska, Colorado, Georgia, Kentucky, Maryland, Massachusetts, Michigan, New Jersey, New Mexico, North Carolina, Ohio, Oklahoma, Oregon, Rhode Island, South Carolina, Utah, Virginia, and Wisconsin.

^†^ Adjusted odds ratios measure the association between the decedent having the demographic or incident characteristic and the race of the decedent being AI/AN. Each adjusted odds ratio used white as the reference group and controlled for age group and sex. Therefore, odd ratios for age groups and sex are not presented.

**^§^** Chi-squared test result for difference between AI/AN and white significant at p<0.05.

^¶^ ZIP Code Rural-Urban Commuting Area (RUCA) codes (2010) were used to determine whether a victim resided in a nonmetropolitan versus a metropolitan area. Victim residential ZIP codes were dichotomized as “metro” (RUCA codes 1–3) and “nonmetro” (RUCA codes 4–10). Descriptions of the RUCA classifications codes 1–10 are available at https://www.ers.usda.gov/data–products/rural–urban–commuting–area–codes/documentation/.

** No significant difference was found between AI/AN and white for this incident characteristic, therefore no measure of association was calculated.

^††^ Statistical reliability criteria for logistic regression not met because cell frequencies were less than the required minimum.

**^§§^** Includes suicides that occurred in a motor vehicle, street, highway, parking lot/garage, public transport, railroad tracks, or bridge.

^¶¶^ Includes suicides that occurred in a beach, river, field, or woods.

*** Includes suicides that occurred in jail, prison, or supervised residential facility.

^†††^ Includes suicides that occurred in industrial or construction sites or an abandoned house, building, or warehouse.

Circumstance information, obtained primarily through information provided by persons who knew the decedent as indicated in coroner/medical examiner reports and law enforcement reports, was known for 87.5% of AI/AN and 89.8% of white suicides (Table 2). Although intimate partner problems were a common precipitating circumstance for both AI/AN (39.1%) and white decedents (29.4%), AI/AN had significantly higher odds of experiencing this circumstance (aOR = 1.2; 95% CI = 1.1–1.3). Approximately two in 10 AI/AN suicides were preceded by an argument, compared with one in 10 white suicides (aOR = 1.4; 95% CI = 1.2–1.7). Compared with white decedents, AI/AN decedents had 2.4 times the odds of the suicide of a friend or family member affecting their death (as ascertained through a note or interviews with persons who knew the decedent) (95% CI = 1.9–3.1) and 1.7 times the odds of the nonsuicide death of a friend or family member affecting their death (95% CI = 1.4–2.1).

**TABLE 2:** Circumstances precipitating suicide deaths of non-Hispanic American Indian/Alaska Natives compared with non-Hispanic whites — National Violent Death Reporting System, 18 states,* 2003–2014

Circumstance	No. (%)^†^		aOR (95% CI)^§^
	AI/AN	White	
**Total decedents**	**1,531 (100)**	**103,986 (100)**	**—**
Cases with known circumstances^¶,^**	1,339 (87.5)	93,403 (89.8)	—
**Suicide event**			
History of suicidal thoughts or plan^††^	111 (33.4)	6,955 (32.7)	—^§§^
History of suicide attempts^¶^	308 (23.0)	18,935 (20.3)	1.0 (0.9–1.2)
Disclosed suicidal intent^¶^	457 (34.1)	26,377 (28.2)	1.3 (1.1–1.4)
**Interpersonal**			
Intimate partner problem^¶^	524 (39.1)	27,464 (29.4)	1.2 (1.1–1.3)
Family relationship problem^¶¶^	83 (10.6)	4,965 (8.8)	—^§§^
Victim of interpersonal violence within past month^¶^	21 (1.6)	444 (0.5)	—***
Perpetrator of interpersonal violence within past month^¶^	91 (6.8)	3,107 (3.3)	2.0 (1.6–2.4)
Argument preceded death^¶,¶¶^	154 (19.7)	6,102 (10.8)	1.4 (1.2–1.7)
**Life stressor**			
Victim in custody^¶,^**	76 (5.0)	2,458 (2.4)	1.7 (1.4–2.2)
Released from institution within previous month^¶,†††^	17 (4.6)	1,885 (8.2)	—***
Criminal legal problem^¶^	201 (15.0)	8,493 (9.1)	1.5 (1.3–1.7)
Civil legal problem^¶^	34 (2.5)	3,420 (3.7)	—***
Physical health problem^¶^	144 (10.6)	21,655 (23.2)	0.9 (0.7–1.0)
Job problem^¶,§§§^	92 (7.6)	12,038 (13.2)	0.5 (0.4–0.6)
Financial problem^¶,§§§^	76 (6.3)	11,211 (12.3)	0.5 (0.4–0.7)
School problem^¶¶¶^	36 (21.4)	688 (22.0)	—^§§^
Eviction/Loss of home	25 (1.9)	2,525 (2.7)	—^§§^
Suicide of friend or family member^¶^	79 (5.9)	1,797 (1.9)	2.4 (1.9–3.1)
Death of friend or family member^¶^	118 (8.8)	6,116 (6.6)	1.7 (1.4–2.1)
Any crisis within past 2 weeks	411 (30.7)	26,815 (28.7)	—^§§^
**Mental health/Substance use**			
Current mental health problem^¶^	371 (27.7)	43,614 (46.7)	0.4 (0.4–0.5)
Current depressed mood^¶^	489 (36.5)	38,940 (41.7)	0.9 (0.8–1.0)
Current mental health treatment^¶^	261 (19.5)	31,987 (34.2)	0.5 (0.4–0.5)
History of mental health treatment^¶^	311 (23.2)	37,499 (40.2)	0.4 (0.4–0.5)
Reported alcohol use in hours preceding death^¶^	651 (48.6)	23,370 (25.0)	2.7 (2.4–3.0)
Alcohol abuse problem^¶^	371 (27.7)	17,242 (18.5)	1.8 (1.6–2.1)
Substance abuse problem other than alcohol	202 (15.1)	14,365 (15.4)	—^§§^

**Abbreviations:** AI/AN = non-Hispanic American Indian/Alaska Native; aOR = adjusted odds ratio; CI = confidence interval; NVDRS = National Violent Death Reporting System; white = non-Hispanic white.

* Alaska, Colorado, Georgia, Kentucky, Maryland, Massachusetts, Michigan, New Jersey, New Mexico, North Carolina, Ohio, Oklahoma, Oregon, Rhode Island, South Carolina, Utah, Virginia, and Wisconsin.

^†^ Denominator includes only those suicides with ≥1 precipitating circumstances, unless otherwise noted. Sums of percentages in columns may exceed 100% because a suicide could have more than one precipitating circumstance.

^§^ Adjusted odds ratios measure the association between the decedent having the precipitating circumstance present and the race of the decedent being AI/AN. Each adjusted odds ratio used white as the reference group and controlled for age group and sex.

^¶^ Chi-squared test result for difference between AI/AN and white significant at p<0.05.

** Denominator includes all suicide decedents (1,531 AI/AN; 103,986 white).

^††^ Variable added to NVDRS in 2013; Denominator includes only decedents from 2013 and later with ≥1 known circumstances (332 AI/AN; 21,246 white).

^§§^ No significant difference was found between AI/AN and white for this incident characteristic, therefore no measure of association was calculated.

^¶¶^ Variable added to NVDRS in 2009; Denominator includes only decedents from 2009 and later with ≥1 known circumstances (780 AI/AN; 56,274 white).

*** Statistical reliability criteria for logistic regression not met because cell frequencies were less than the required minimum.

^†††^ Variable added to NVDRS in 2013; Denominator includes all suicide decedents from 2013 and later (367 AI/AN; 22,959 white). Institution includes jail or other detention facility, hospital, psychiatric institution, supervised residential facility or nursing home.

^§§§^ Denominator includes only decedents ≥18 years with ≥1 known circumstance (1,213 AI/AN; 91,097 white).

^¶¶¶^ Denominator includes only decedents ≤18 years with ≥1 known circumstance (168 AI/AN; 3,125 white).

Current diagnosed mental health problems (aOR = 0.4; 95% CI = 0.4–0.5), depressed mood (aOR = 0.9; 95% CI = 0.8–1.0), and current mental health treatment (aOR = 0.5; 95% CI = 0.4–0.5) were less likely to be reported among AI/AN decedents than among white decedents (Table 2). Substance abuse problems other than alcohol were not significantly different between AI/AN and white decedents; however, AI/AN decedents had 1.8 times the odds of a reported alcohol problem compared with white decedents (95% CI = 1.6–2.1). In addition, AI/AN decedents were more likely to have reportedly used alcohol in the hours before death (aOR = 2.7; 95% CI = 2.4-3.0) and had more than twice the odds of a positive alcohol toxicology result (aOR = 2.1; 95% CI = 1.9–2.5) (Table 3). Among those tested, AI/AN decedents were significantly more likely to test positive for marijuana (aOR = 1.5; 95% CI = 1.2–1.8) and amphetamines (aOR = 1.4; 95% CI = 1.1–1.9), and significantly less likely to test positive for antidepressants (aOR = 0.7; 95% CI = 0.5–0.9) and opioids (aOR = 0.5; 95% CI = 0.4–0.7) than were white decedents (Table 3).

**TABLE 3:** Toxicology* results of non-Hispanic American Indian/Alaska Native suicide decedents compared with non-Hispanic white suicide decedents — National Violent Death Reporting System, 18 states,^†^ 2003–2014

Toxicology	AI/AN		White		aOR (95% CI)^§^
	No. (%) tested	No. (%) positive	No. (%) tested	No. (%) positive	
Alcohol	846 (55.3)	449 (53.5)	66,955 (64.4)	23,436 (35.0)	2.1 (1.9–2.5)
Amphetamine	593 (38.7)	47 (8.0)	42,762 (41.1)	1,966 (4.7)	1.4 (1.1–1.9)
Antidepressant	389 (25.4)	77 (20.2)	39,489 (38.0)	11,329 (28.7)	0.7 (0.5–0.9)
Benzodiazepine	148 (9.7)	23 (15.8)	11,142 (10.7)	4,003 (36.1)	^—¶^
Cocaine	607 (39.7)	24 (4.0)	45,757 (44.0)	2,786 (6.1)	^—¶^
Marijuana	481 (31.4)	98 (20.7)	35,374 (34.0)	3,802 (10.9)	1.5 (1.2–1.8)
Opioid	614 (40.1)	72 (11.7)	46,773 (45.0)	11,126 (24.1)	0.5 (0.4–0.7)

**Abbreviations:** AI/AN = non-Hispanic American Indian/Alaska Native; aOR = adjusted odds ratio; CI = confidence interval; white = non-Hispanic white.

* All substances included in the table had a chi-squared test results that was significant at p<0.05. Substances indicating no significant difference between AI/AN and white at p<0.05 were excluded from the table.

^†^ Alaska, Colorado, Georgia, Kentucky, Maryland, Massachusetts, Michigan, New Jersey, New Mexico, North Carolina, Ohio, Oklahoma, Oregon, Rhode Island, South Carolina, Utah, Virginia, and Wisconsin.

^§^ Adjusted odds ratios measure the association between the decedent having tested positive for the substance and the race of the decedent being AI/AN. The denominator was the number of decedents who were tested for each substance. Each adjusted odds ratio used white as the reference group and controlled for age group and sex.

^¶^ Statistical reliability criteria for logistic regression not met because cell frequencies were less than the required minimum.

## Discussion

Suicide rates among AI/AN are historically higher than those of the total U.S. population (*1*). The results of this study are consistent with previous research on risk factors for AI/AN suicidal behaviors (*3*,*5*) and provide additional information on important circumstances and characteristics that precede suicide among AI/AN. Across many demographics, incident characteristics, and circumstances, AI/AN decedents were significantly different from whites.

Approximately 70% of AI/AN decedents resided in nonmetropolitan areas, including rural settings, underscoring the importance of implementing suicide prevention strategies in rural AI/AN communities. Residential status can affect the circumstances surrounding suicide. For example, in this study AI/AN decedents had lower odds than did white decedents of having received a mental health diagnosis or mental health treatment, even when controlling for age and sex. Rural areas often have lower availability and use of mental health services because of provider shortages^††^ and social barriers, including stigma and lack of culturally competent care (*6*). To address provider shortages, financial incentives, such as loan forgiveness for mental health practitioners, represent one strategy that could be helpful in recruiting providers for rural and nonmetropolitan areas (*2*). The high rate of suicides among AI/AN youths highlights the need for early prevention. School-based programs are able to reach a large number of AI/AN youths at high risk and could increase the availability of services for AI/AN in isolated nonmetropolitan areas (*4*). In addition, school-based programs that focus on individual life skills development and interpersonal social emotional learning programs to promote healthy relationships and conflict resolution might address the higher occurrence of intimate partner problems and arguments preceding AI/AN suicides (*2*,*4*).

AI/AN decedents were more likely to have a friend’s or family member’s suicide contribute to their death. A previous study in one AI/AN tribe found that suicidal behavior occurred close in time and within tight social networks, suggesting suicide contagion (*5*). Given the observation that AI/AN had an elevated risk of their own suicide being linked to the suicide death of a loved one, community level prevention strategies, including programs that focus on postvention (e.g., survivor support groups) and safe reporting of suicides by the media (e.g., not using sensationalized headlines), should be considered (http://reportingonsuicide.org/wp-content/themes/ros2015/assets/images/Recommendations-eng.pdf) (*2*).

Substance use is a recognized risk factor for suicidal behavior (*4*). A larger proportion of AI/AN decedents used alcohol before their suicide and had reported alcohol abuse problems. Previous studies have found that AI/AN youths aged 12–17 years have the highest rates of alcohol use among all racial/ethnic groups (*4*). Community-based programs to reduce excessive alcohol use (e.g., enforcement of laws prohibiting sales to minors and increasing alcohol taxes) and individual-level programs for persons at various risk levels, such as improved access to substance abuse treatment and life skills development programs for youths are necessary (*1*,*4*,*7*). Differences in the prevalence of alcohol use, interpersonal problems, and access to mental health treatment among AI/AN might be symptoms of disproportionate exposure to poverty, historical trauma, and other contexts of inequity and should not be viewed as inherent to AI/AN culture (*4*,*8*).

The findings in this report are subject to at least five limitations. First, race of AI/AN decedents is often misclassified on death certificates resulting in underascertainment of AI/AN mortality, including suicide (*9*). Second, tribal affiliation is not collected in NVDRS. Thus, the heterogeneity of AI/AN tribes and the cultural differences between these communities could not be assessed, and results might not be generalizable across all AI/AN communities. Future studies are needed to identify risk and protective factors for suicide that might be unique to individual tribes or communities. Third, it was not possible to determine whether decedents resided on tribal reservations based on the available information. Fourth, mental health diagnoses and treatment status are based on informant reports and could be underreported for either or both groups. Finally, NVDRS data were available from 18 states as of the time of this report and are therefore not necessarily representative of suicides outside these areas.

Prior research suggests comprehensive suicide prevention strategies designed to address the specific needs of an AI/AN community are associated with reductions in suicide (*10*). The high prevalence of suicide among AI/AN and the comparative differences in suicide circumstances among this group are illustrative of the inequities faced by this population. This study highlights the importance of focused suicide prevention and intervention efforts that incorporate culturally relevant, evidence-based strategies at the individual, interpersonal, and community levels (*2*).

**Summary**

**What is already known about this topic?**

American Indian/Alaska Natives (AI/AN) have the highest rates of suicide of any racial/ethnic group in the United States. The rates of suicide in this population have been increasing since 2003.

**What is added by this report?**

Analysis of National Violent Death Reporting System data from 18 states showed AI/AN suicide decedents were younger and had higher odds of living in a nonmetropolitan area than did non-Hispanic whites who died by suicide. Suicide and nonsuicide deaths of friends and family, as well as alcohol use preceding death were associated with AI/AN decedents more often than non-Hispanic white decedents.

**What are the implications for public health practice?**

The high prevalence of suicide among the AI/AN population and the comparative differences in suicide circumstances among AI/AN decedents illustrate some of the disparities this population faces. Focused, yet comprehensive, suicide prevention and intervention efforts are needed that incorporate culturally relevant, evidence-based strategies at the individual, interpersonal, and community levels.

## Footnotes

* https://wonder.cdc.gov/ucd-icd10.html.

† In 2003, the National Violent Death Reporting System (NVDRS) began data collection with six states (Maryland, Massachusetts, New Jersey, Oregon, South Carolina, and Virginia) participating; seven states (Alaska, Colorado, Georgia, North Carolina, Oklahoma, Rhode Island, and Wisconsin) joined in 2004, three (Kentucky, New Mexico, and Utah) in 2005, and two (Michigan and Ohio) in 2010. Ohio collected statewide data starting in 2011 and Michigan starting in 2014. CDC provides funding for state participation, and the ultimate goal is for NVDRS to expand to include all 50 states, U.S. territories, and the District of Columbia.

§ https://www.cdc.gov/violenceprevention/pdf/nvdrs_web_codingmanual.pdf.

¶ Information on race and ethnicity are recorded as separate items in NVDRS consistent with U.S. Department of Health and Human Services (HHS) and Office of Management and Budget standards for race/ethnicity categorization. HHS guidance on race/ethnicity is available at https://aspe.hhs.gov/datacncl/standards/ACA/4302/index.shtml.

** ZIP Code Rural-Urban Commuting Area (RUCA) codes (2010) were used to determine whether decedents resided in nonmetropolitan versus metropolitan areas. RUCA codes measure daily commuting flows, population density, and urbanization levels to classify sub-county level geographic areas. Victim residential ZIP codes were dichotomized as “metro” (RUCA codes 1–3) and “nonmetro” (RUCA codes 4–10). Descriptions of the RUCA classification codes 1–10 are available at https://www.ers.usda.gov/data-products/rural-urban-commuting-area-codes/documentation/.

†† https://datawarehouse.hrsa.gov/topics/shortageAreas.aspx.
